# Economic Impact of Hospitalization Past Maximal Neurosurgical Inpatient Benefit

**DOI:** 10.7759/cureus.3567

**Published:** 2018-11-10

**Authors:** Cara M Rogers, Christopher M Busch, Joshua A Cuoco, Zev Elias, Gary R Simonds

**Affiliations:** 1 Neurosurgery, Carilion Clinic-Virginia Tech Carilion School of Medicine, Roanoke, USA

**Keywords:** inpatient healthcare costs, economic waste, disposition delays, neurosurgery, socioeconomics, healthcare system, healthcare improvement

## Abstract

Background

The unsustainable cost of healthcare in the United States has made it important for all healthcare professionals to examine their practices for wasteful spending and work to mitigate these costs. When neurosurgical patients remain hospitalized beyond the point of maximum inpatient benefit, this represents a potential source of healthcare economic waste.

Objective

The objective of this study was to determine the direct and indirect costs to a hospital system when neurosurgical patients remain hospitalized past the maximum inpatient benefit and identify targets to improve this potential wasteful spending.

Methods

We performed an extensive chart review of all patients admitted to our neurosurgical service from the months of July to October 2016, who had been deemed medically stable for discharge but remained in the hospital past their ideal date of discharge. We analyzed for significant trends in patient factors, procedural acuity, disposition, funding, and other factors that contributed to the delays in discharge.

Results

A total of 334 patients were admitted to the Carilion Clinic-Virginia Tech Carilion (CC-VTC) inpatient neurosurgery service, and 50 of these admissions (15%) resulted in medically unnecessary prolonged hospitalizations. These patients were hospitalized for a total of 324 days past the dates of ideal discharge. Elective cases had the maximum number of prolonged hospitalizations, while the emergent cases had the maximum number of prolonged hospitalization days. Patients with private insurance had the shortest number of prolonged hospitalization days, and uninsured patients had the longest. Patients requiring disposition to a rehabilitation or a nursing facility remained in the inpatient setting for longer periods than those destined for home. The most common factors limiting appropriate discharge were related to bed availability at outside facilities, funding issues, and differing opinions on appropriate disposition. The medically unnecessary days accounted for 41% of the total hospitalization but accounted for only 12.9% of the billable charges. The billable cost per medically necessary day was $17,326 in comparison to a medically unnecessary day of $2,070. Indirect costs were inferred from these patients utilizing beds and resources that could have been allocated to others with acute needs, given that our hospital is at capacity and on diversion, a significant percentage of the time.

Conclusion

Neurosurgical patients remaining hospitalized past their maximal inpatient benefit have a significant economic impact on a hospital system. Identifying patients who are at risk for prolonged hospitalizations may provide us with the targets for improvement to mitigate this healthcare economic waste.

## Introduction

With healthcare costs reaching unsustainable levels, it is imperative that we identify sources of wasteful spending and implement changes to eliminate this waste. Hospital costs constitute the largest component of healthcare spending and are therefore a prime target for improvement. A potential source of healthcare economic waste exists when patients remain hospitalized after they have reached the “maximum inpatient benefit.” This is a commonly encountered occurrence during our neurosurgical service at Carilion Clinic-Virginia Tech Carilion (CC-VTC). The goal of our study was to determine the financial impact of inappropriately prolonged hospitalizations on our neurosurgical service. We also aimed to identify the potential targets for improvement.

## Materials and methods

We tracked all patients admitted to the CC-VTC neurosurgical service from the months of July to October 2016. Patients who were admitted to our neurosurgical service but subsequently transferred to the care of another service were excluded from the study. The census was reviewed by the rounding attending physician and team on a daily basis. Patients who were deemed medically stable for discharge, but remained in the hospital, were determined to have reached the “maximum inpatient benefit,” and were included in the study. These patients were considered to have medically unnecessary prolonged hospitalization days. 

Each patient who qualified for inclusion was then studied through a chart review. The date of ideal discharge was considered the date the patient was medically stable for discharge. The date of actual discharge was the date that the patient was eventually discharged from the hospital. The total days of hospitalization were calculated from the date of admission to the date of discharge. The total days of medically unnecessary hospitalization were calculated as the difference between the ideal and actual dates of discharge. 

We recorded patient demographics including age, gender, acuity of presentation, primary payer status, and disposition destination. The acuity of presentation was recorded as elective, urgent, or emergent. Elective cases were defined as admissions scheduled in advance for non-emergent procedures. Urgent cases were defined as admissions for procedures to preserve the patient’s life but did not require emergent surgery. Emergent cases were defined as admissions for emergency surgery. The primary payer status was categorized as medicare, medicaid, private insurance, or uninsured. Disposition destination was categorized as home, inpatient rehabilitation, or nursing facility. Home disposition was defined as patients being discharged to their private residence with or without home health services. Inpatient rehabilitation was defined as any facility intended for a provisional stay and required patients to participate in therapies for at least three hours daily. Nursing facilities were defined as institutions without minimum participation requirements for short or long-term admission. Each case was also analyzed for the primary barriers to appropriate discharge.

A one-way analysis of variance was conducted to determine if statistical significance was present between the acuity of presentation, the primary payer status, or the type of disposition and the number of prolonged hospitalization days. For groups that demonstrated statistical significance, a Tukey’s honestly significant difference test was conducted to determine the specific subgroups that exhibited statistical difference.

Economic impact was measured as direct and indirect costs to the hospital system. To determine the direct cost, a cost analysis was performed by the Director of Cost Accounting for each patient included in the study. We determined the billable costs for the entire hospitalization and the billable costs for the days of medically unnecessary hospitalization. The indirect economic impact was considered as any negative effect of having patients fill beds unnecessarily, thereby limiting space for other admissions. 

## Results

During the four-month period between July 1 and October 31, 2016, a total of 334 patients were admitted to the CC-VTC neurosurgical inpatient service at the Roanoke Memorial Hospital. This included outpatient elective admissions and patients admitted from the emergency room. Fifty patients, or 15% of these admissions, resulted in medically unnecessary prolonged hospitalizations. There was a female predominance in the cohort with 58% females and 42% males. The range of patient ages was from 20 to 90 years of age with the average patient age being 63 years. The details of these cases are listed in Table 1. These 50 patients were hospitalized for a total of 784 days, of which 324 days were past the dates of ideal discharge. The average length of total hospitalization was 15.68 days, with the average length of prolonged hospitalization being 6.48 days. Therefore, 41% of the days that these patients were hospitalized were deemed medically unnecessary.

**Table 1:** Details of cases that resulted in prolonged days of stay SNF: skilled nursing facility; IPR: in-patient rehabilitation

Neurosurgical Procedure	Age	Sex	Prolonged Days	Acuity	Payer Status	Disposition Destination
Craniotomy for clipping of a ruptured aneurysm	37	Female	27	Emergent	Self-pay	SNF
Coiling of a ruptured aneurysm	56	Female	12	Emergent	Private	IPR
Craniotomy for clipping of a ruptured aneurysm	38	Female	14	Emergent	Medicaid	IPR
Coiling of a ruptured aneurysm	38	Female	7	Emergent	Private	Home
Craniotomy for subdural hematoma evacuation	74	Female	6	Emergent	Medicare	IPR
Craniotomy for clipping of a ruptured aneurysm	61	Female	5	Emergent	Private	IPR
Biopsy of the vertebral body of a pathologic compression fracture	50	Female	5	Urgent	Private	Home
Cranial wound exploration for wound dehiscence	63	Female	8	Urgent	Private	SNF
Craniotomy for tumor resection	66	Female	2	Urgent	Medicare	IPR
Anterior-posterior cervical fusion for trauma	60	Female	3	Urgent	Private	IPR
Stereotactic needle biopsy of an intracranial lesion	90	Female	3	Urgent	Medicare	Home
T7-L1 fusion for trauma	59	Female	2	Urgent	Private	Home
Craniotomy for resection of tumor	68	Female	2	Urgent	Medicare	IPR
Craniotomy for resection of tumor	55	Male	7	Urgent	Private	IPR
Burr hole evacuation of acute on chronic subdural hematoma	90	Female	10	Urgent	Medicare	IPR
Craniotomy for resection of tumor	68	Female	6	Urgent	Medicare	IPR
Craniotomy for resection of tumor	48	Male	2	Urgent	Medicaid	IPR
Cranial wound exploration for wound infection/dehiscence	54	Male	7	Urgent	Medicaid	SNF
Craniotomy for resection of tumor	47	Male	2	Urgent	Medicaid	SNF
Craniotomy for resection of tumor	56	Female	1	Urgent	Self-pay	Home
Cranial wound exploration for pseudomeningocele	58	Male	4	Urgent	Medicaid	SNF
Craniotomy for resection of tumor	76	Male	20	Urgent	Medicare	IPR
Cranioplasty	49	Male	6	Elective	Private	IPR
Cranioplasty	63	Female	4	Elective	Private	IPR
Anterior-posterior cervical fusion	81	Female	7	Elective	Medicare	Home
Re-admitted for pain control status-post cervical fusion	62	Male	12	Elective	Medicaid	IPR
Craniotomy for resection/fenestration of cyst	54	Male	3	Elective	Medicare	SNF
Craniotomy for clipping of an unruptured aneurysm	65	Male	8	Elective	Private	IPR
L3-5 decompression and repair of cerebrospinal fluid leak	84	Female	7	Elective	Medicare	SNF
Cranioplasty	20	Male	20	Elective	Medicaid	IPR
Craniotomy for clipping of an unruptured aneurysm	62	Male	9	Elective	Medicaid	SNF
Craniotomy for resection of a cerebellopontine angle tumor	65	Female	22	Elective	Medicare	IPR
Cervicothoracic decompression and fusion	73	Male	2	Elective	Medicare	IPR
L3-5 transforaminal lumbar interbody fusion	55	Female	3	Elective	Medicaid	Home
Ventriculoperitoneal revision	70	Female	8	Elective	Medicare	IPR
C3-5 posterior decompression	63	Male	5	Elective	Private	IPR
C3-6 anterior cervical decompression and fusion	55	Male	2	Elective	Self Pay	IPR
L2-S1 decompression and fusion	64	Female	1	Elective	Private	SNF
Cranioplasty	79	Female	2	Elective	Medicare	IPR
L4-5 decompression	83	Female	4	Elective	Medicare	SNF
L4-5 decompression	76	Male	3	Elective	Medicare	IPR
Resection of posterior cervical paraspinal mass	75	Male	4	Elective	Medicare	Home
Revision C1-6 posterior fusion	76	Female	3	Elective	Medicare	SNF
Craniotomy for clipping of an unruptured aneurysm	53	Female	4	Elective	Medicaid	Home
C3-6 anterior cervical decompression and fusion	77	Male	2	Elective	Medicare	SNF
C5-T1 posterior decompression and fusion	74	Male	2	Elective	Private	SNF
L4-5 transforaminal lumbar interbody fusion	60	Female	5	Elective	Medicaid	Home
L4-5 transforaminal lumbar interbody fusion	73	Male	15	Elective	Medicare	SNF
Craniotomy for clipping of an unruptured aneurysm	66	Female	2	Elective	Medicare	Home
L2-S1 decompression	62	Male	4	Elective	Private	SNF

Evaluation of the acuity of presentation in this cohort demonstrated that the elective cases had the greatest number of prolonged hospitalizations, while the emergent cases had the maximum prolonged hospitalization days. Of the 50 patients included in the study, there were six emergent, 16 urgent, and 28 elective cases. Elective cases accounted for 56% of prolonged hospitalizations. The elective patients remained in the hospital an average of 6.00 days past their ideal dates of discharge (Table 2). Urgent cases accounted for 32% of the prolonged hospitalizations and had an average of 5.25 days of medically unnecessary hospitalization. Emergent cases accounted for 12% of the prolonged hospitalizations and had an average of 11.83 days of medically unnecessary hospitalization. There was a significant effect of neurosurgical acuity on the number of prolonged hospitalization days at the *p *< 0.05 level for the three conditions [F (2,47) = 3.285, *p* = 0.046]. Post-hoc comparisons using the Tukey’s honestly significant difference test indicated statistical significance between the emergent and urgent groups (*p* = 0.043) but did not demonstrate statistical significance between the emergent and elective groups (*p* = 0.062) or the elective and urgent groups (*p* = 0.894).

**Table 2:** Prolonged days of stay based on acuity of presentation

Acuity	Patients	Prolonged Days	Average Prolonged Days
Emergent	6	71	11.83
Urgent	16	84	5.25
Elective	28	169	6.00

Analysis of the primary payer status revealed that patients with private insurance had the shortest prolonged hospitalization days, and uninsured patients had the longest. Of the 50 patients included in the study, 15 had private insurance, 21 had medicare, 11 had medicaid, and three were uninsured. The average length of prolonged hospitalization was 5.27 days for patients with private insurance, 6.33 days for medicare patients, 7.45 days for medicaid patients, and 10.00 days for uninsured patients (Table 3). No statistical significance was observed between the type of insurance and the number of prolonged hospitalization days (*p* = 0.567).

**Table 3:** Prolonged days of stay based on payer status

Payer Status	Patients	Prolonged Days	Average Prolonged Days
Self Pay	3	30	10.00
Medicaid	11	82	7.45
Medicare	21	133	6.33
Private	15	79	5.27

The length of medically unnecessary prolonged hospitalization also varied with the disposition plan. Patients requiring disposition to a rehabilitation or a nursing facility remained in the inpatient setting for a longer period than those destined for a private residence. Of the 50 patients included in the study, 11 were discharged home, 15 to nursing facilities, and 24 to inpatient rehabilitation. The average length of prolonged hospitalization was 3.91 days for patients discharged home, 6.53 days for patients discharged to a nursing facility, and 7.63 days for patients discharged to inpatient rehabilitation (Table 4). No statistical significance was observed between the type of disposition and the number of prolonged hospitalization days (*p* = 0.215).

**Table 4:** Prolonged days of stay based on disposition destination

Disposition Destination	Patients	Prolonged Days	Average Prolonged Days
Home	11	43	3.91
SNF	15	98	6.53
IPR	24	183	7.63

The barriers we encountered to discharging patients on their ideal dates of discharge were multifactorial in most cases. The most common factors limiting appropriate discharge were related to bed availability at outside facilities, funding issues, and differing opinions on appropriate disposition. Limited bed availability at inpatient rehabilitation and nursing facilities contributed to a delay in discharge in 54% of cases. Awaiting insurance authorization for inpatient rehabilitation and nursing facilities contributed to a delay in discharge in 20% of cases. A lack of adequate funding or benefits for inpatient rehabilitation and nursing facilities contributed to delays in 22% of cases. Multiple insured patients did not have benefits or had run out of their benefit for rehabilitation or nursing facility placement. Disagreement regarding appropriate disposition contributed to a delay in discharge 32% of cases. This discrepancy was oftentimes between our inpatient therapy consultant recommendations and the outpatient facility assessment of the patient needs. This included cases where the patients were felt to be too high functioning or too low functioning for disposition to the intended facility. 

The direct cost of these medically unnecessary prolonged hospitalizations was determined through a cost analysis by the Director of Cost Accounting. The total billable charges for the hospitalizations of the 50 patients included in the study were $6,124,676. The total billable charges for the medically unnecessary days past the ideal dates of discharge were $474,230. The medically unnecessary days accounted for 41% of the total hospitalization but accounted for only 12.9% of the billable charges. The billable cost per medically necessary day was calculated to be $17,326 in comparison to the billable cost per medically unnecessary day being $2,070.

**Figure 1:**
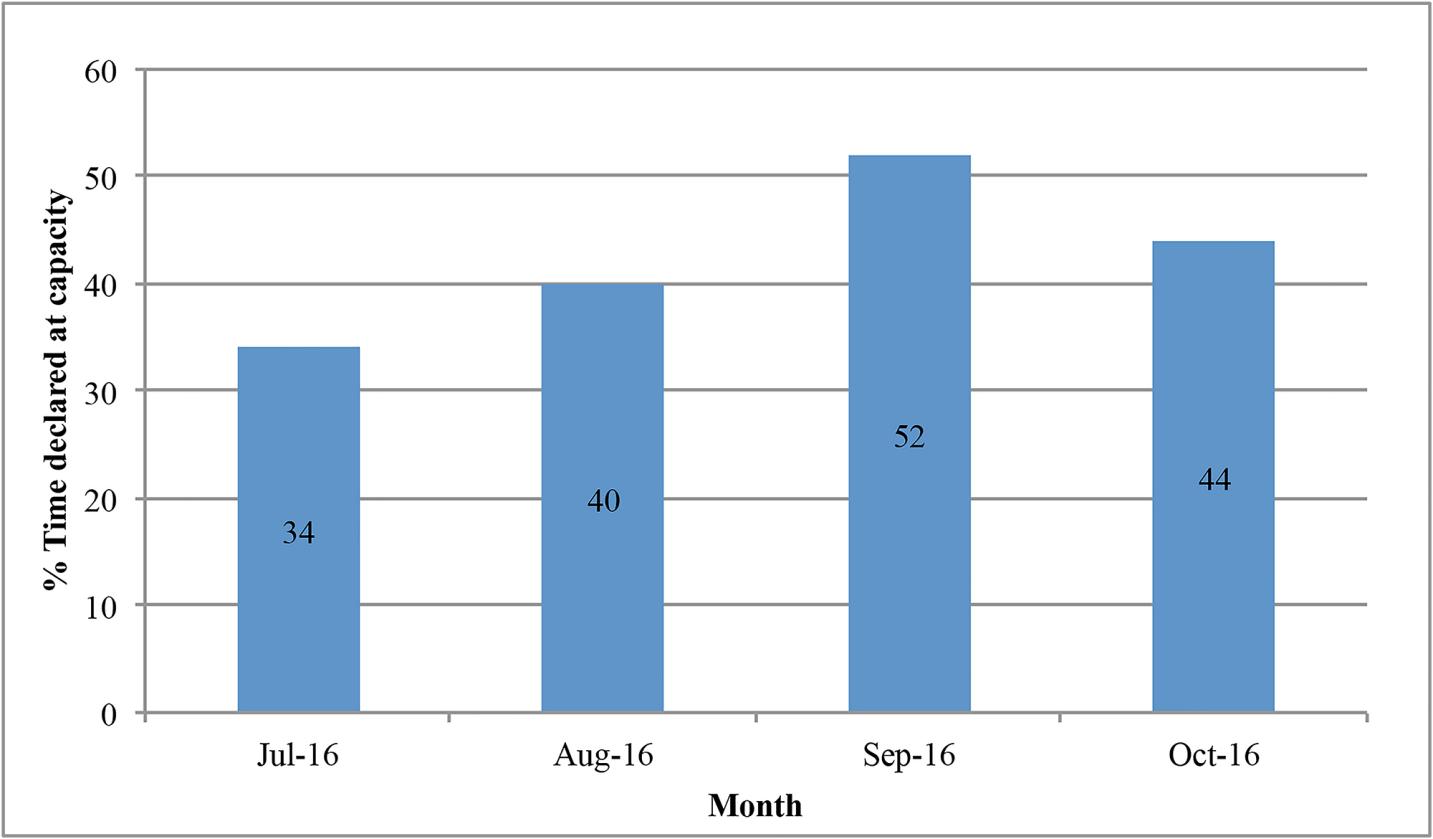
Percentage of time at declared capacity per shift

**Figure 2:**
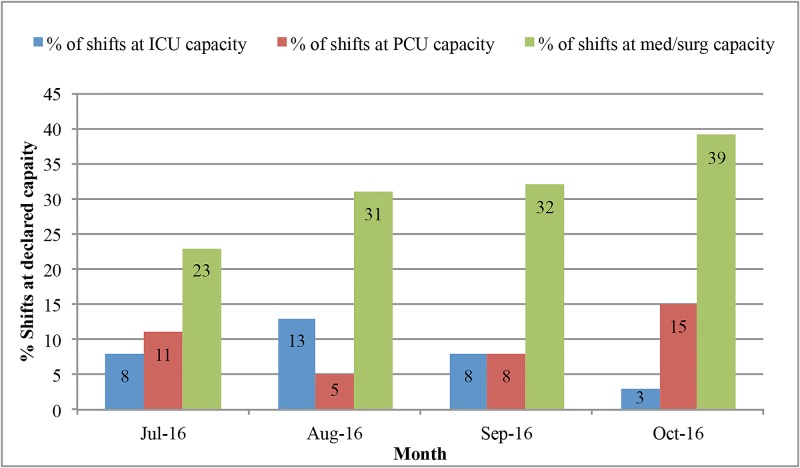
Percentage of shifts at declared capacity by the level of care ICU: intensive care unit; PCU: progressice care unit

The indirect costs to the hospital system were not financially measurable but were inferred from the fact that these patients were filling hospital beds that could have been utilized for other patients. In general, our system remains at or near capacity for inpatient beds. Our hospital was on some level of diversion 42.5% of the shifts during this four-month period (Figure 1). The hospital is unable to accept transfers from the referring facilities or direct admissions from the outpatient setting when we are at capacity for the requested level of patient care. The patients included in this study were taking up medical/surgical unit floor beds during the medically unnecessary days of prolonged hospitalization. The medical/surgical floor beds were filled to capacity 31.25% of shifts on average during these months (Figure 2). Our emergency room also goes onto diversion when there is an increase in patient volumes that exceed the available resources. Our emergency room was on diversion a total of 238 hours in these four months, which is almost 10 full days or 8% of the time. 

## Discussion

The cost of healthcare in the United States has risen to an unsustainable level, which has forced us to evaluate the efficiency of utilization of these resources. Healthcare costs have been on a steady rise, accounting for 17.8 percent of the gross domestic product in 2015 with projections that it will continue to rise [1-2]. The national healthcare expenditure increased 5.8% that year to $3.2 trillion dollars with the hospital expenditures increasing 5.6% to $1 billion dollars [1,3]. However, despite spending almost twice as much per capita on health care as other industrialized nations, according to the Census Bureau, the US ranks 42^nd^ in the world for life expectancy and has higher rates of infant mortality, obesity, and avoidable deaths than the global averages [4]. Therefore, even though the government continues to allocate a substantial and an increasing portion of our resources to healthcare, there has been no compensatory improvement in outcomes according to the Organization for Economic Cooperation and Development [5].

Concern exists that an unacceptable portion of healthcare spending is unnecessary, inefficient, or underutilized [4,6-7]. It was calculated by Pricewaterhouse Coopers in 2008 that 1.2 trillion dollars, over half of the healthcare costs that year, was wasteful spending [8]. They defined healthcare waste as “costs that could have been avoided without a negative impact on quality” [8]. In order to eliminate this waste, it is essential for providers to identify sources of this wasteful spending. Inpatient hospital costs are of particular importance because they make up the largest component of healthcare spending and represent a prime target for improvement.

Our neurosurgical team at CC-VTC in Roanoke, Virginia, identified medically unnecessary prolonged hospitalizations as a potential source of wasteful spending. In a four-month period, 15% of the patients admitted to our service had medically unnecessary prolonged hospitalizations. The average length of prolonged hospitalization was 6.48 days and accounted for 41% of the days that these patients were hospitalized. The average billable cost per medically unnecessary day was $2,070 which compounds on itself for every day that patients remain hospitalized needlessly. This resulted in $474,230 of billable costs that were unnecessary and did not improve the quality of care. 

While this represents a wasteful utilization of resources with respect to the healthcare system, the impact on the hospital system is also substantial. There are predetermined bundled costs for patients being admitted for a particular neurosurgical procedure after which the services that a hospital can charge for diminishes precipitously regardless of disposition delays. This explains why the billable cost for each medically necessary day is over eight times greater than the cost for each medically unnecessary day. The financial impact of that difference is likely great. The hospital was on some level of diversion 42.5% of the time, and the emergency room was on diversion 8% of the time during this four-month period. Therefore, while patients remained in our hospital beds past their ideal dates of discharge, this limited our ability at times to bring in patients with acute medical needs. While the hospital system is only able to charge a comparatively small amount of money per medically unnecessary day, they are losing out on a greater potential revenue from these diverted new admissions. This represents a potential loss of profit for the hospital system and also a potential delay in care for patients. 

We were able to identify the potentially preventable causes of prolonged hospitalizations that represent targets for improvement. Notably, we found that 56% of prolonged hospitalizations occurred in our elective admissions. In 50% of these cases, there were delays due to the lack of bed availability at the rehabilitation or nursing facility of the patients choosing. In 22% of these cases, there were delays while we awaited insurance authorization for these facilities. This may be preventable by identifying patients who are unlikely to be discharged home and setting up their disposition prior to admission. This could include determining the insurance benefits they have, preference of facilities, bed availability at these facilities, and securing insurance authorization. Patients admitted for urgent procedures would likely benefit from this preadmission process as well. Patients admitted for emergent procedures should have social workers involved early in their admissions to identify the potential barriers to discharge. This would include identifying legal decision makers, funding sources, and viable discharge possibilities. We also identified that there is a shortage of beds at the rehabilitation and nursing facilities in our region, resulting in delays in discharge in 54% of the cases. It may, therefore, be beneficial for our hospital system to work toward increasing the available beds at the current facilities or increase the number of facilities in the region.

## Conclusions

Our results confirmed that unnecessarily prolonged hospitalizations on a neurosurgical service have a significant impact on the medical system. It is a prime example of unnecessary and inefficient utilization of resources to keep patients hospitalized without inpatient medical needs. It contributed to significant bed shortages, which forces us to turn away patients with medical needs. By identifying the commonly encountered barriers to appropriate discharge, we have now discovered several targets for improvement.
